# Emergence and Genomic Characterization of a KPC-2-, NDM-1-, and IMP-4-Producing *Klebsiella michiganensis* Isolate

**DOI:** 10.3389/fmicb.2021.762509

**Published:** 2022-01-06

**Authors:** Yanyan Zhang, Danxia Gu, Xuemei Yang, Yuchen Wu, Congcong Liu, Zhangqi Shen, Rong Zhang

**Affiliations:** ^1^Department of Clinical Laboratory, Second Affiliated Hospital, Zhejiang University School of Medicine, Hangzhou, China; ^2^Department of Clinical Laboratory, Zhejiang Provincial People’s Hospital, Hangzhou, China; ^3^Department of Infectious Diseases and Public Health, Jockey Club College of Veterinary Medicine and Life Sciences, City University of Hong Kong, Kowloon, Hong Kong SAR, China; ^4^Beijing Advanced Innovation Center for Food Nutrition and Human Health, College of Veterinary Medicine, China Agricultural University, Beijing, China

**Keywords:** *Klebsiella michiganensis*, antibiotic resistance, KPC, NDM, IMP

## Abstract

A rectal swab sample was collected from a patient with Guillain–Barré syndrome and enriched in lysogeny broth. Carbapenem-resistant bacteria were selected by China Blue agar plates containing 0.3 μg/ml meropenem. Carbapenemase-producing *Klebsiella michiganensis* was identified and characterized by matrix-assisted laser desorption/ionization time-of-flight (MALDI-TOF), immune colloidal gold technique, a conjugation experiment, PCR analysis, and antimicrobial susceptibility testing. The genome of *K. michiganensis* was determined by whole genome sequencing. Antimicrobial susceptibility testing showed that the *K. michiganensis* was resistant to imipenem, meropenem, ertapenem, cefmetazole, ceftazidime, cefotaxime, piperacillin/tazobactam, sulbactam/cefoperazone, ceftazidime/avibactam, cefepime, and aztreonam while susceptible to polymyxin B, ciprofloxacin, tigecycline, and amikacin. Immune colloidal gold technique suggested that this strain co-produced three different carbapenemases [*Klebsiella pneumoniae* carbapenemase (KPC), New Delhi metallo-beta-lactamase (NDM), and Imipenem (IMP)]. Whole genome sequencing analysis indicated that this strain belonged to ST91, and *bla*_KPC–2_, *bla*_NDM–1_, and *bla*_IMP–4_ were carried on different conjugative plasmids. Besides, the co-existence and transferability of *bla*_KPC–2_, *bla*_NDM–1_, and *bla*_IMP–4_ in *K. michiganensis* facilitates the potential horizontal dissemination and nosocomial spread of resistance genes among multidrug-resistant organisms.

## Introduction

*Klebsiella michiganensis*, first recovered from a toothbrush holder in a Michigan household in 2013, was reported to be closely related to *Klebsiella oxytoca* with similarity of 16S rRNA sequence as high as 99% (Saha et al., 2013). Since then, this pathogen has been increasingly recognized as an emerging human pathogen and associated with nosocomial infections (Hazen et al., 2018;

Zheng et al., 2018; Seiffert et al., 2019; Chapman et al., 2020). In 2018, a *K. michiganensis* isolate co-producing KPC-2, NDM-1 and NDM-5 was identified in a Chinese patient with acute diarrhea (Zheng et al., 2018). Later, a case of bloodstream infection caused by KPC-3-producing *K. michiganensis* was reported in Switzerland (Seiffert et al., 2019). In South Africa, an OXA-181 and NDM-1-producing *K. michiganensis* was characterized from a stool sample of a cancer patient (Founou et al., 2018). Recently, a bla_*VIM*–1_-carrying *K. michiganensis* isolate from the rectal swab of a Turkish patient was identified in Switzerland (Campos-Madueno et al., 2021). As mentioned above, *K. michiganensis* containing various kinds of carbapenemases genes has been sporadically reported in recent years, which could be a reservoir for the spread of these important resistance genes to other pathogens. Since the protein spectrum from *K. michiganensis* was close to *K. oxytoca*, it is always identified as *K. oxytoca* by matrix-assisted laser desorption/ionization time-of-flight mass spectrometry (MALDI-TOF MS) (Saha et al., 2013; Founou et al., 2018; Seiffert et al., 2019; Chapman et al., 2020). Based on the phenotypic, biochemical, chemotaxonomic, and molecular differences between *K. michiganensis* and *K. oxytoca*, the former was identified successfully, although the differences were small (Saha et al., 2013). And *K. michiganensis* was also identified based on average nucleotide identity (ANI) in reports from recent years (Zheng et al., 2018; Seiffert et al., 2019).

During a study to evaluate carbapenem-resistant strains from rectal swabs in 2021, an isolate of *K. oxytoca* identified by MALDI-TOF MS carrying KPC-2, NDM-1, and IMP-4 was detected, and later this isolate was reidentified as *K. michiganensis* according to whole genome sequencing (WGS) analysis. We described its genomic and phenotypic features in this report.

## Case Report

Rectal swabs for carbapenem-resistant Enterobacterales (CRE) screening were collected in 2021 from patients admitted to the Second Affiliated Hospital of Zhejiang University (SAHZJU) with 2,200 beds and located in Hangzhou, Zhejiang Province, China. Strain K210011 was obtained from a 44-year-old male patient who was hospitalized with “weakness of the extremities deteriorating after 7 days and loss of speech after 3 days” and isolated from China Blue Agar (CBA) plates containing 0.3 μg/ml meropenem after overnight incubation at 37°C. Strain K210011, initially identified as *K. oxytoca* by MALDI-TOF MS (MicroIDSys, shanghai, China), was reconfirmed as *K. michiganensis* according to the Kleborate result based on the WGS data (Lam et al., 2021).

Antimicrobial susceptibility testing (AST) was performed by broth microdilution; the minimum inhibitory concentration (MIC) of tigecycline was interpreted according to European Committee on Antimicrobial Susceptibility Testing (EUCAST) breakpoints, and the other MICs were interpreted using CLSI-M100 standard [Jiayue et al., 2020; Clinical and Laboratory Standards Institute [CLSI], 2021; The European Committee on Antimicrobial Susceptibility Testing [EUCAST], 2021]. As shown in Table 1, *K. michiganensis* K210011 was resistant to all the cephalosporins tested (including cefmetazole, ceftazidime, and cefepime), to the combinations with β-lactamase inhibitors (piperacillin/tazobactam, cefoperazone/sulbactam, and the novel combination ceftazidime/avibactam), and to carbapenems. This isolate was susceptible to polymyxin B, ciprofloxacin, tigecycline, and amikacin. According to NG-Test ^®^ CARBA 5 (zhongshengzhongjie, Changsha, China), K210011 was positive for Imipenem (IMP), *Klebsiella pneumoniae* carbapenemase (KPC), and New Delhi metallo-beta-lactamase (NDM).

**TABLE 1:** Characteristics of K210011 and corresponding transconjugants.

Strain	Gene	MIC (mg/L)														
		IMP	MEM	ETP	CMZ	CAZ	CTX	TZP	SCF	CAV	FEP	PB	TGC	CIP	AK	ATM
K210011	*bla*_IMP–4_ bla_KPC–2_ bla_NDM–1_	4	16	64	>128	>128	>128	>256/4	>256/128	>64/4	>64	1	≤0.25	≤1	≤4	64
EC600	/	≤ 1	≤1	≤2	≤ 2	≤2	≤4	≤ 8/4	≤8/4	≤ 0.5/4	≤4	≤0.5	≤ 0.25	≤1	≤4	≤ 4
TC-1	*bla*_KPC–2_ *bla*_NDM–1_	2	2	8	16	>128	64	128/4	128/64	>64/4	16	≤ 0.5	≤0.25	≤ 1	≤4	32
TC-2	*bla*_NDM–1_ *bla*_IMP–4_	4	8	64	>128	>128	128	64/4	>256/128	>64/4	64	≤ 0.5	≤0.25	≤ 1	≤4	≤4
TC-3	*bla*_IMP–4_ *bla*_KPC–2_ *bla*_NDM–1_	8	8	16	>128	>128	>128	256/4	>256/128	>64/4	>64	≤ 0.5	≤0.25	≤ 1	≤4	64

*IMP, imipenem; MEM, meropenem; ETP, ertapenem; CMZ, cefmetazole; CAZ, ceftazidime; CTX, cefotaxime; TZP, piperacillin/tazobactam; SCF, sulbactam/cefopcrazone; CAV, ceftazidime/avibactam; FEP, cefepime; PB, polymyxin B; TGC, tigecycline; CIP, ciprofloxacin; AK, amikacin; ATM, aztreonam; TC-1, TC-2, TC-3, transconjugants of K210011.*

Conjugation assay was performed by filter-mating as previously reported (Eckert et al., 2006). Selected transconjugants showed six different resistance gene profiles (three contained a single resistance gene, and three harbored at least two genes). All transconjugants carried more than a single plasmid. It remains unclear which plamids are self-conjugative and which plasmids are mobilizable. Further experiments would be needed to confirm the functionality of the tra gene complexes sequenced. Among these, three representative transconjugants (named TC-1, TC-2, and TC-3), which contained at least two types of resistance genes, were further characterized. The three transconjugants showed a similar resistance phenotype (Table 1). Corresponding resistance genes were verified by PCR and sequencing as described previously (Dallenne et al., 2010; Nordmann et al., 2011b). Distinct resistance genes were detected among the three strains; strain TC-1 was positive for blaKPC-2 and blaIMP-4 located on pK210011_KPC and pK210011_IMP, respectively. Strain TC-2 carried blaNDM-1 and blaIMP-4 located on pK210011_NDM and pK210011_IMP, respectively. Strain TC-3 was positive for all of the three carbapenemase genes located on their corresponding plasmids.

Genomic DNA of strain K21001 was extracted using the PureLink Genomic DNA Mini Kit (Invitrogen, Carlsbad, CA, United States), then subjected to whole-genome sequencing *via* the 150-bp pair-end Illumina HiSeq X10 platform and also subjected to the long-read Oxford Nanopore Technologies MinION platform after being treated with a supplementary sequencing kit (Nanopore, Oxford, United Kingdom). Both short and long reads were *de novo* hybrid assembled using Unicycler v0.4.8 (Wick et al., 2017). Genome sequences were annotated with RAST (Overbeek et al., 2013) and Prokka (Seemann, 2014). The size of strain K210011 was 7,007,106 bp (base pairs), including a 5.71-Mb chromosome and five plasmids with the sizes of 320,473, 246,963, 233,442, 121,353, and 101,156 bp. K210011 was found to belong to ST91 based on multilocus sequence typing (MLST) by BIGSdb (Jolley et al., 2018). This strain did not harbor virulence genes common in *Klebsiella pneumoniae* isolates, such as yersiniabactin, colibactin, aerobactin, salmochelin, *rmpA*, or *rmpA2* by Kleborate (Lam et al., 2021). Searched against the resistance gene database by ResFinder 2.1 (Zankari et al., 2012), strain K210011 was found to harbor the resistance genes *sul1*, *bla*_OXY–5–2_, *bla*_NDM–1_, *bla*_KPC–2_, and *bla*_IMP–4_, with *bla*_OXY–5–2_ being located on the chromosome.

**FIGURE 1:**
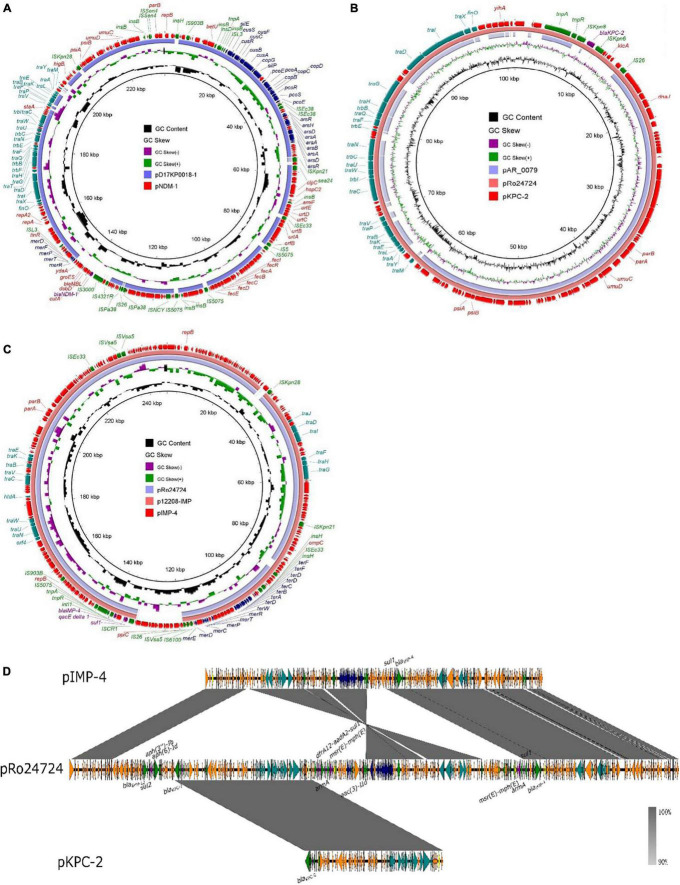
Alignment of plasmid pK210011_NDM, pK210011_KPC and pK210011_IMP. **(A)** Plasmid pK210011_NDM showed highest similarity (88% coverage and 100% identity) to plasmid pD17KP0018-l (GenBank accession no. CP052337.1). **(B)** Plasmid p K210011_KPC showed highest similarity (100% coverage and 99.96% identity) to plasmid pRo24724 (GenBank accession no. CP021328.1) and plasmid pAR_0079 (GenBank accession no. CP029000.1, 84% coverage and 99.98% identity). **(C)** Plasmid pK210011_IMP showed highest similarity (93% coverage and 100% identity) to plasmid p12208-IMP (GenBank accession no. MF344562.1) and plasmid pRo24724 (92% coverage and 99.90% identity). **(D)** Alignment of plasmid pK210011_KPC, pK21001 l_IMP and pRo24724.

According to PlasmidFinder (Carattoli et al., 2014), the gene *bla*_NDM–1_ was located on the IncFIB(K)/IncFII(K) 233,442-bp plasmid, designated as pK210011_NDM (Figure 1A). According to BLAST, plasmid pK210011_NDM showed highest similarity (88% coverage and 100% identity) to plasmid pD17KP0018-1 (GenBank accession no. CP052337.1), a 233,970-bp plasmid recovered from *K. pneumoniae*. The *bla*_KPC–2_ gene was located on the IncFIB(pQil) 101,156-bp plasmid, designated as pK210011_KPC (Figure 1B). Plasmid pK210011_KPC showed highest similarity (100% coverage and 99.96% identity) to plasmid pRo24724 (GenBank accession no. CP021328.1), a 446,611-bp plasmid recovered from *Raoultella ornithinolytica*. The highest identity was to a plasmid from *K. pneumoniae*, the 125,913-bp plasmid pAR_0079 (GenBank accession no. CP029000.1) with an 84% coverage and 99.98% identity, which did not harbor the gene *bla*_KPC–2_. The gene *bla*_IMP–4_ was found to be located in the 246,963-bp plasmid designated as pK210011_IMP (Figure 1C). This plasmid also harbored the *sul1* gene. Plasmid pK210011_ IMP showed the highest similarity (93% coverage and 100% identity) to plasmid p12208-IMP (GenBank accession no. MF344562.1), a 323,333-bp plasmid from *K. pneumoniae* and plasmid pRo24724 (92% coverage and 99.90% identity). As plasmids pK210011_KPC and pK210011_IMP both resembled plasmid pRo24724, we aligned these plasmids together. The result indicated that plasmids pK210011_KPC and pK210011_IMP were part of the large plasmid pRo24724 and might have evolved from pRo24724-like plasmid (Figure 1D). Alignment and visualization of plasmids was conducted with the BLAST Ring Image Generator (BRIG) (Alikhan et al., 2011). Insertion sequences (ISs) were identified using ISfinder and ISsaga (Siguier et al., 2006). Assembled genome sequences were submitted to the National Center for Biotechnology Information (NCBI) database with accession number JAHNZR000000000.

## Discussion

Carbapenem-resistant Enterobacteriaceae isolates are a significant threat to public health. In the current study, we report the first clinical *K. michiganensis* isolate (to the best of our knowledge) harboring *bla*_*KPC*–2_, *bla*_*NDM*–1_, and *bla*_*IMP*–4_ located on different conjugative plasmids. The carbapenemases NDM-1 and its variants are the third most common carbapenemases after KPC and oxacillinases (OXA) (Wu et al., 2019) in Asian countries especially in China (Farhat and Khan, 2020). KPC is the primary carbapenemase in Enterobacteriaceae, and KPC-2 and KPC-3 are the most frequently observed variants (Doi and Paterson, 2015). IMP is one of the most common variants of class B metallo-β-lactamases (MBLs) and was first discovered in *Serratia marcescens* in Japan. Since then, MBLs have been reported in several parts of world (Nordmann et al., 2011a). KPC, OXA, and NDM-type enzymes frequently appear in *K. pneumoniae* and *Escherichia coli* isolates (Han et al., 2020; Kazmierczak et al., 2021). *K. michiganensis* is an uncommon gram-negative bacterium in the clinical environment, first recovered from a toothbrush holder in Michigan, America, in 2013. Since then, clinical cases of infections caused by carbapenem-resistant *K. michiganensis* isolates are frequently reported (Founou et al., 2018; Hazen et al., 2018; Zheng et al., 2018; Seiffert et al., 2019; Chapman et al., 2020; Campos-Madueno et al., 2021). *K. michiganensis* has been brought to the attention of the public as an emerging human pathogen associated with nosocomial infections. In the current study, we only obtained transconjugants carrying more than one single plasmid; thus, we speculate on the possibility of (co-)transfer of resistance genes, which needs to be verified by additional experiments in the future. We also highlighted the accumulation of resistance genes in rarely identified *K. michiganensis*, so we should monitor this emerging human pathogen to minimize the problems with this pathogen. However, monitoring of *K. michiganensis* is hampered by a possible misidentification as *K. oxytoca*, because of the similarity of protein spectra between *K. michiganensis* and *K. oxytoca*. Therefore, further research is needed to develop precise, simple, and specific identification methods.

## Data Availability Statement

The datasets presented in this study can be found in online repositories. The names of the repository/repositories and accession number(s) can be found below: https://www.ncbi.nlm.nih.gov/, JAHNZR000000000.

## Ethics Statement

The studies involving human participants were reviewed and approved by this study was approved by the Ethics Committee of Second Affiliated Hospital, Zhejiang University School of Medicine (2018-039). The subjects gave written informed consent in accordance with the Declaration of Helsinki. The patients/participants provided their written informed consent to participate in this study. The animal study was reviewed and approved by Ethics Committee of Second Affiliated Hospital, Zhejiang University School of Medicine (2018-039).

## Author Contributions

YZ conducted the research and wrote the manuscript. DG and ZS revised the manuscript. XY analyzed the plasmids. YW and CL collected the samples. RZ designed the study. All authors contributed to the article and approved the submitted version.

## Conflict of Interest

The authors declare that the research was conducted in the absence of any commercial or financial relationships that could be construed as a potential conflict of interest.

## Publisher’s Note

All claims expressed in this article are solely those of the authors and do not necessarily represent those of their affiliated organizations, or those of the publisher, the editors and the reviewers. Any product that may be evaluated in this article, or claim that may be made by its manufacturer, is not guaranteed or endorsed by the publisher.
